# Overexpression of *SikRbcs2* gene promotes chilling tolerance of tomato by improving photosynthetic enzyme activity, reducing oxidative damage, and stabilizing cell membrane structure

**DOI:** 10.1002/fsn3.1631

**Published:** 2020-05-25

**Authors:** Li Zhang, Jing Yang, Xinyong Guo, Aiying Wang, Jianbo Zhu

**Affiliations:** ^1^ Key Laboratory of Agricultural Biotechnology College of Life Science Shihezi University Shihezi China; ^2^ Agricultural Product Quality Safety Supervision and Management Station Wuwei China

**Keywords:** chlorophyll fluorescence, low‐temperature tolerance, photosynthesis, *Saussurea involucrata*, *SikRbcS2*, tomato

## Abstract

Red blood cell is a small subunit encoding 1, 5‐ribulose bisphosphate carboxylase/ oxygenase (Rubisco). It could control the catalytic activity of Rubisco and play an important role in plant photosynthesis. *SikRbcs2*, a small subunit of Rubisco, is cloned from *Saussurea involucrate*. It has a strong low‐temperature photosynthetic and photorespiration ability, but its mechanism in cold tolerance remains to be unknown. The results of quantitative PCR showed that *SikRbcS2* gene could be induced by low‐temperature, osmosis, and salt stress. Its expression was increased with the decrease of temperature, which was consistent with the habitat of *Saussurea involucrata*. Overexpression of *Sikrbcs2* could significantly increase the mRNA expressions of SlrbcL and SlRCA in transgenic tomato seedlings. Furthermore, the activity and content of Rubisco and Rubisco activase (RCA) in transgenic tomato seedlings were also significantly higher than those in wild‐type plants. The contents of chlorophyll and carotenoids, soluble sugar, and starch in the leaves of transgenic plants were significantly higher than those in WT plants, as well as the plant height, leaf area, and dry matter weight. Moreover, compared with WT, MDA content was decreased, and activities of SOD, POD, CAT, and APX were significantly higher in transgenic lines. In conclusion, our results suggested that overexpression of *SikRbcs2* can reduce the damage of low temperature on photosynthesis of tomato seedlings. It could help achieve relatively stable photosynthesis, enhance scavenging ROS ability of tomato seedlings, maintain stable membrane structure, and improve cold tolerance of tomato.

## Graphical abstract

The excessive expression of SikRbcs2 can reduce the damage of low temperature to photosynthetic mechanism of tomato seedlings, maintain relatively stable photosynthesis, enhance the ability of ROS scavenging of tomato seedlings, maintain stable membrane structure, and improve cold tolerance of tomato.

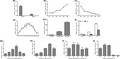

## 1 INTRODUCTION

1, 5‐ribulose bisphosphate carboxylase/oxygenase (Rubisco) is at the circular intersection of plant photosynthetic carbon reduction and photosynthetic carbon oxidation (Portis 1990,2003). As a key enzyme of photosynthetic carbon assimilation, it has a decisive effect on net photosynthetic rates (Andersson & Backlund, 2008; Spreitzer & Salvucci, 2002). Rubisco is made up of eight large subunits (RbcL) and eight small subunits (RbcS). RbcL plays a catalytic role while RbcS has the function of regulating Rubisco activity (Portis, Li, Wang, & Salvucci, 2007). Previous studies found that RbcS gene was suitable for the study of plant molecular evolution and phylogeny (Feller, Crafts‐Brandner, & Salvucci, 1998; Sage, Way, & Kubien, 2008). RbcS gene promoter is an important tool for the specific expression of exogenous genes in the leaves of recipient plants in genetic engineering (Suzuki et al., 2007). RbcS is essential to the structure of enzyme activity, which plays an important role in the ratio of shuttle to oxidation (Whitney & Andrews, 2001). In addition, it can improve the photosynthetic capacity of recipient plants through transplanting exogenous RbcS genes into recipient plants or enhancing the gene expression of RbcS (Furbank, Chitty, von Caemmerer, & Jenkins, 1996). This exogenous RbcS gene may also be involved in the plant's antisignaling system and play an important role in growth, maturation, and senescence of plants. RbcL of spinach Rubisco has been splicing with the transporter genes of RbcS of tomato (Lam & Zechman, 2006; Van Oosten & Besford, 1994). This chimeric gene has been integrated into tobacco, so that the transgenic tobacco can grow normally. Transgenic arabidopsis thaliana only expresses RbcS, and the expression of RbcS in transgenic arabidopsis thaliana is significantly higher than WT. Rubisco activity and Ru‐BP content would not decrease under low light intensity.

Tomatoes (*Lycopersicon esculentum*) belong to cold sensitive plant. In the winter, they often encounter low‐temperature stress, resulting in the decrease of photosynthesis, and the decrease of yield and quality (Zhang et al., 2011). Rubisco is a speed‐limiting enzyme for photosynthesis (Ngernprasirtsiri, Kobayashi, & Akazawa, 1988). RbcS, which regulates the expression of RbcL, is an essential part of enzyme activity structure (Fritz, Wolter, Schenkemeyer, Herget, & Schreier, 1993). Therefore, cloning and genetic transformation of RbcS gene are of great significances to explore the effect of RbcS overexpression on growth, development, and photosynthesis of tomato at molecular level. *Sasussured involucrata* has response and adaptation mechanism to low‐temperature stress and improves photosynthetic capacity at low temperature (Wang et al., 2007). However, studies on the role of Rubisco in *Sasussured involucrata* and its regulation mechanism of photosynthesis have not been reported. Therefore, the purpose of this study was to investigate the adaption mechanism of tomatoes to low‐temperature stress with the assistance of *SikRbcs2*.

## 2 MATERIALS AND METHODS

### 2.1 Materials

*Saussurea involucrata* plants were cultured in our laboratory at 21°C/19°C, with a photoperiod of 16 hr light/8 hr dark and a light intensity of 13 K lux. Tomato (*Lycopersicon esculentum*) variety was Yaxin 87‐5. Genetic transformation receptor in this study was provided by Yaxin Seed Co. Ltd.

*S. involucratas* seedlings were kept in a 20°C light incubator for 3 days and kept in a 20°C dark incubator for another 3 days. Then, *S. involucratas* seedlings were transferred to the dark for 24 hr. *S. involucratas* seedlings, which continued to be dark for 3 days, were also transferred to the light for 24 hr. For cold treatment, the *S. involucratas* seedlings cultured at 20°C were used as control. *S. involucratas* seedlings were treated at different temperatures in the order of 10°C, 4°C, and −2°C for 6 hr each time. *S. involucratas* tissue culture seedlings were immersed in 20% PEG and 150 mmol NaCl for 24 hr. The leaves of *S. involucratas* seedlings were treated at different times, frozen with liquid nitrogen for 5 min, and then stored at −70°C.

WT tomato and those two transgenic tomatoes growing at the age of 2 months were transferred into an artificial climate chamber (light intensity, 70 µmol/m^2^/s; temperature, 25°C/25°C; photoperiod, 12 hr/12 hr; relative humidity, 60%–70%). After 2 days of adaptation, they were treated by low‐temperature stress with temperature gradient of 16°C, 8°C, and 4°C for 5 days and treated at 4°C for 7 days. The experiment was repeated 3 times, with 20 nontransgenic and transgenic tomato plants each time.

### 2.2 Isolation of full‐length *SikRbcS2* cDNA

A full‐length cDNA library from *S. involucrata* was constructed using the Creator™ SMART™ cDNA Library Construction Kit (Clontech). Ninety‐six single clones were randomly selected and sequenced. One cDNA sequence showed homology to a gene which encoded the Rubisco RbcS gene. This cDNA sequence was named *SikRbcS2*. The 5′ end, CDS region, and 3′ end of *SikRbcS2* gene were identified using ORF finder and were aligned with known sequence databases. We also confirmed the ORF of *SikRbcS2* gene by prokaryotic expression (data not shown). A phylogenetic tree was plotted using DNAMAN and MEGA4.1 software based on amino acid sequences from the NCBI database.

### 2.3 RNA extraction and RT‐qPCR analysis

Total RNA was isolated from different treatment and control samples separately using RNAiso Plus kit (TaKaRa) with on‐column DNase I treatment. According to the *S. involucrata GAPDH* gene (accession no. KF563904.1), the reference gene, primers GAPDHF1 and GAPDHR1, Primers SikRbcS2F1 and SikRbcS2R2 were designed based on the SikRbcS2 gene sequence (Table 1). Using reverse‐transcribed cDNA as a template, GAPDHF1, GAPDHR1 and SikRbcS2F1, and SikRbcS2R2 were used as primers following the instructions of the SYBR Green I Master Mix kit. Amplification was performed using a Light Cycler^®^ 480II PCR machine (Roche). Each sample was repeated 3 times, and the data were analyzed by the 2^−ΔΔCT^ method.

**TABLE 1:** Primers used in this study

primers	Sequences
*GAPDHF1*	5'‐ TTCAACATTATTCCCAGCAGCAC−3'
*GAPDHR1*	5'‐ TAAGTAGCCTTCTTCTCAAGTCTCACA−3'
*SikRbcS2 F1*	5' ‐GCCTCCGCTCAAGCCAACAT−3'
*SikRbcS2 R1*	5'‐CTGAACTCTTCCACCGTTGCTG−3'

### 2.4 Plasmid construction and tomato transformation

To obtain the Pro35S: *SikRbcS2* construct, SikRbcS2 cDNA was amplified using the forward primer 5′‐CCATGGAGTTATCAGTCGACGGTACGGGA‐3′ (underline indicates *Nco*I) and the reverse primer 5′‐ GGTAACCCGCCAACGAATGGTCTAG AAAGC ‐ 3′ (underline indicates *BstE*II). The PCR product was digested with *Nco*I and *BstE*II, and ligated into a pCAMBIA1301 vector double digested with the same enzymes. To obtain the ProRD29A: *SikRbcS2* construct, the RD29A promoter was first amplified using the forward primer 5′‐AAGCTTCGACTCAAAACAA ACTTACGAA‐3′ (underline indicates *Hind*III site) and the reverse primer 5′‐CCATGGAATCAAACCCTTTATTCCTGA‐3′ (underline indicates *Nco*I site), and was cloned into the pCAMBIA1301 vector. The construct was then digested with *Nco*I and *BstE*II and ligated to the *SikRbcS2* PCR product digested with the same enzymes. The identity of clone insert was confirmed by sequencing. These two constructs were introduced into *Lycopersicon esculentum* WT plants (Yaxin 87‐5) via *Agrobacterium tumefaciens*‐mediated (strain GV3101) T‐DNA transformation. Transformants were selected on MS medium (Sigma‐Aldrich) containing 80 μg/l of kanamycin and then transferred to soil to set seeds. Kanamycin‐resistant T1 seedlings were confirmed by RT‐PCR using primers of *SikRbcS2*. Almost all seedlings from independent transgenic T1 lines were survived with MS medium containing 80 mg/L kanamycin. These were then transferred to soil to set seed. The T2 generation transgenic seeds were germinated in flower pots at 25°C in 60%‐70% humidity, with a light intensity of 70 µmol/m^2^/s and a 12 hr light/12 hr dark cycle.

### 2.5 Measurements of chlorophyll fluorescence and photosynthetic parameters

Net photosynthetic rates (Pn), stomatal conductance (Gs), transpiration rate (Tr), and intercellular CO_2_ concentration (Ci) were measured in the second apical leaves using portable photosynthetic system (Ciras‐2, PP Systems International). Constant PFD (600 μmol/m^2^/s), CO_2_ concentration (350–360 mg/L), and leaf temperature (25 ± 1°C) were maintained throughout all measurements. Each measurement was repeated at least three times. Fluorescence was measured at 25°C with five replicates using a portable fluorometer (FMS‐2; Hansatech). Plants were adapted for 20 min in dark before measurement of maximal fluorescence (Fm), variable fluorescence (Fv), initial fluorescence (Fo), coefficient of photochemical quenching (qP), the coefficient of photochemical quenching (qN), and electron transport rate (ETR) in darkness. Maximal photochemical efficiency of photosystem II (PSII) in darkness: Fv/Fm = (Fm − Fo)/Fm.

### 2.6 Measurement of chlorophyll pigment content

Chlorophyll pigment content was measured according to the following procedure. Leaves of WT tobacco and transgenic tobacco (0.1 g) were obtained after treating by different temperatures, respectively. Leaves were cut into filaments of 1 mm wide and added to test tubes. 15 ml of acetone ethanol mixture (V: V = 1:1) was then added, and the extraction was performed in dark until the filaments were completely white at room temperature. The supernatant was mixed and used to test absorbance values at 470, 645, and 663 nm, respectively. The contents of chlorophyll a (Chla), chlorophyll b (Chlb), and carotenoids (Car) were calculated according to those values.

### 2.7 Determination of growth index of tomato overexpressing *SikRbcs2* gene

Leaf growth index (PI) was calculated by PI = n + (In *n*‐In R)/ (In Ln‐In Ln + 1). The reference leaf length *R* is 180 mm, *n* is the number of leaves whose blade length is longer than the reference leaf length, and In Ln and In Ln + 1 are the lengths of the *n*th and *n* + 1 leaves, respectively. Plant height was measured with a ruler, stem was measured with a vernier caliper, and fresh weight and dry weight were measured with an electronic balance. They were measured before treatment, after 12 days of continuous treatment at low‐temperature gradient, and after 14 days of recovery.

### 2.8 Measurement of physiological indices

The malondialdehyde (MDA) content was determined by the modified thiobarbituric acid reaction outlined by Du *et al*. using a spectrophotometer (UV‐160A, Shimadzu Scientific Instruments). Briefly, leaves excised from the tomato plants were washed in deionized water. Leaf disks were punched out, and membrane damage was quantified by measuring MDA concentration. Relative electrolyte leakage (REL) was determined by using an EC 215 Conductivity Meter (Markson Science Inc.) with the method of Du *et al*. The youngest fully expanded leaves were randomly selected and subjected to electrolyte leakage analysis using conductivity meter. The relative conductance was calculated using the formula: REL = (C1 − CW)/(C2 − CW) × 100. C1 is the electrical conductivity value during the first measurement, C2 is the conductivity value after boiling, and CW is the conductivity of deionized water.

### 2.9 Antioxidant enzymes and reactive oxygen species

After exposure to cold and drought treatments, 0.5 g samples of fresh T_2_ WT and transgenic tomato plant leaves were collected. These leaves were cut into pieces and homogenized in an ice bath with 4 ml of 50 mmol/L sodium phosphate buffer (pH 7.8) containing 1% polyvinylpyrrolidone and 10 mmol/L β‐mercaptoethanol. The homogenate was transferred to a tube and centrifuged (18,894.2 *g*) for 15 min at 4°C. The supernatant fluid was then used for the determination of enzyme activity. Ascorbate peroxidase (APX) activity was determined as the decrease in absorbance of ascorbate at 290 nm. The activity of catalase (CAT) was determined. We assessed the activity of superoxide dismutase (SOD) with the light absorption value at 560 nm. Peroxidase (POX) activity was determined. Absorbance was recorded with an Infinite M200 Pro microplate reader (Tecan Group Ltd., Männedorf).

H_2_O_2_ and O_2_^−^ contents were determined according to a standard curve. Absorption values were recorded at 415 and 530 nm using a spectrophotometer (UV‐160A, Shimadzu Scientific Instruments).

### 2.10 Determination of Rubisco and Rubisco Activase (RCA)

Plant activating enzyme ELISA kit (Ta Ka Ra) and plant ribulose‐1,5‐bisphosphate carboxylase/oxygenase ELISA kit (Ta Ka Ra) adopted following the instructions. The absorbance was measured at 450 nm using a microplate reader (M200pro). Sample RCA and Rubisco concentration were calculated by standard curve.

## 3 RESULTS

### 3.1 Isolation and characterization of full‐length SikRbcS2 cDNA in *S. involucrata*

**FIGURE 1:**
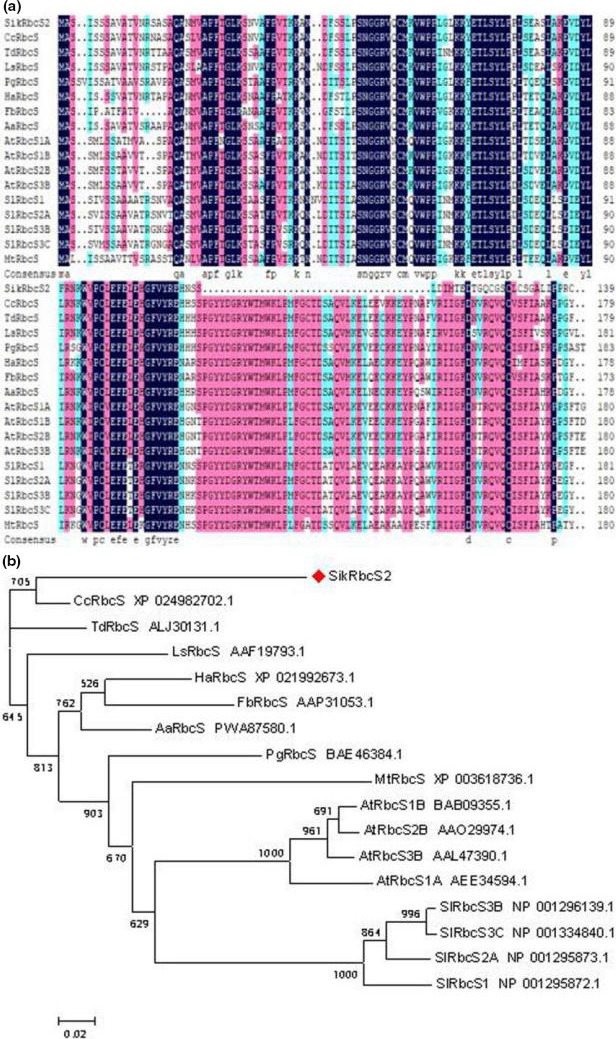
Alignments of the *SikRbcS2* amino acid sequence with proteins from other species. (a) Alignment of the putative SikRbcS2 protein with dehydrin homologs; (b) Phylogenetic tree based on homologous RbcS proteins. The following proteins were included in the alignment: *SikRbcS2* (*Saussurea involucrata*), *CcRbcS* (XP_024982702.1), *TdRbcS* (ALJ30131.1), *LsRbcS* (AAF19793.1), *HaRbcS* (XP_021992673.1), *PgRbcS* (BAE46384.1), *FbRbcS* (AAP31053.1), *AaRbcS* (PWA87580.1), *MtRbcS* (XP_003618736.1), *AtRbcS1A*(AEE34594.1), *AtRbcS1B*(BAB09355.1), *AtRbcS2B*(AAO29974.1), *AtRbcS3B* (AAL47390.1), *SlRbcS1* (NP_001295872.1), *SlRbcS2A* (NP_001295873.1), *SlRbcS3B* (NP_001296139.1), and *SlRbcS3C* (NP_001334840.1)

Full‐length libraries were constructed using the Switching Mechanism at the 5′ end of RNA Transcript (SMART) method. Facilitate the preliminary mapping of transcription start sites due to the high percentage of full‐length clones. Previously, we selected ten clones for sequencing, all of which were revealed to be full‐length cDNAs. To further evaluate the quality of our libraries and identify interest genes, ninety‐six randomly selected single clones were sequenced, from which the full‐length *SikRbcS2* gene was obtained and subsequently confirmed by sequencing. According to DNAMAN (http://us.expasy.org/tools/protparam.html), the full‐length cDNA of the *SikRbcS2* gene is 904 bp in length and it possesses a 420 bp open reading frame (ORF). Analysis using transmembrane helix prediction (TMpred) revealed no transmembrane helices in the deduced SikRbcS2 protein sequence, suggesting that the SikRbcS2 protein may have no significant role in the membrane and it must play a role in other places (e.g., in the cytosol or nucleus). Homology analysis of the deduced protein sequence was then performed using Phyre (http://www.sbg.bio.ic.ac.uk/phyre/). Next, we discovered that 139 amino acid residues of the SikRbcS2 protein were highly similar (61.45% identity) with a protein from *Cynara cardunculus var. scolymus* by using NCBI BLASTP (Figure 1a). As shown in the phylogenetic tree (constructed using MEGA), the SikRbcS2 protein shared the closest genetic relationship with an RbcS protein from *Cynara cardunculus var. scolymus* (Figure 1b).

### 3.2 Analysis of expression pattern of *SikRbcS2* in *S. involucrata* under different conditions

**FIGURE 2:**
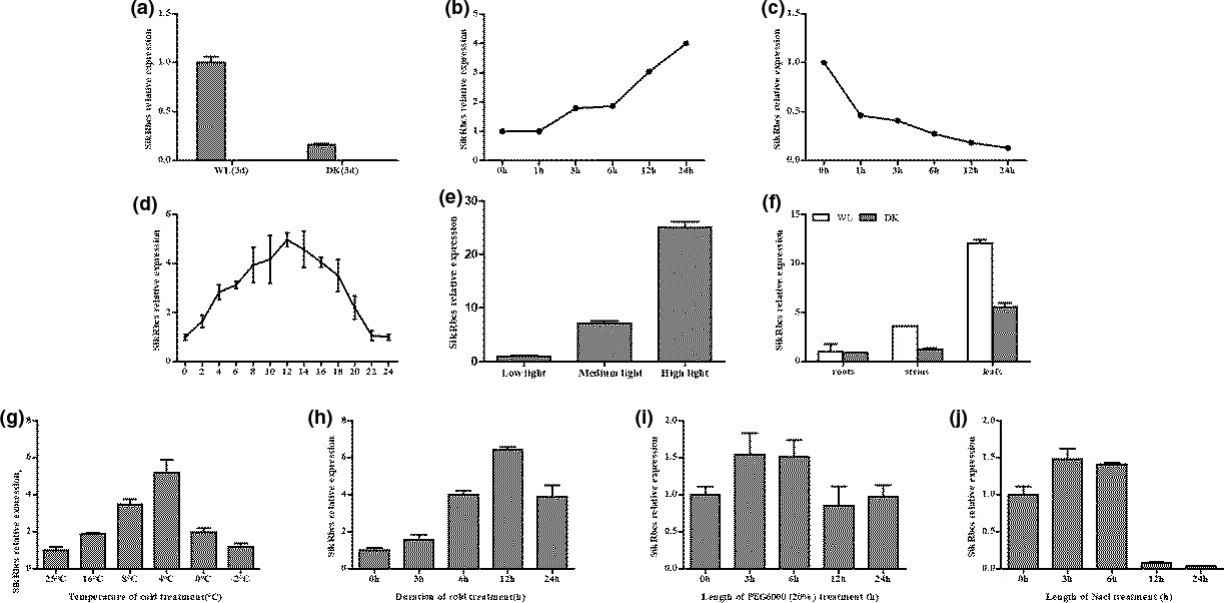
Relative expression levels of *SikRbcS2* under different conditions analyzed by RT‐qPCR. (a) under continuous white light or darkness; (b) under the condition from darkness to white light; WL: under white light, Dk: under darkness; (c) under the condition from white light to darkness; WL: under white light, Dk: under darkness; (d) under the condition of 16 hr light and 8 hr darkness during 24 hr; (e) under different light intensities; weak light: 1.257 μmol/m^2^/s, medium light: 42.7 μmol/m^2^/s, intense light: 1,235 μmol/m^2^/s; (f) in different organs of *S. involucrata*. *GAPDH* was used as the control. (g) *SikRbcS2* expression in *S. involucrata* under low‐temperature treatment (25°C, 16°C, 8°C, 4°C, 0°C, −2°C) for 2 hr. (h) *SikRbcS2* expression in *S. involucrata* under low‐temperature treatment (4°C) for 0, 3, 6, 12, and 24 hr. (i) *SikRbcS2* expression in *S. involucrata* under simulated drought stress (20% PEG6000) for 0, 3, 6, 12, and 24 hr. (j) *SikRbcS2* expression in *S. involucrata* under 150 mmol NaCl stress for 0, 3, 6, 12, and 24 hr

In order to study the function of promoter, the *S. involucrata* seedlings were treated with light, low‐temperature, simulated drought, and salt stress according to the relevant functional elements of the sequence. And tissue expression specificity of *SikRbcS2* in the seedlings was detected. Materials treated under different conditions were collected to extract their total RNA, and the relative expression levels of *SikRbcS2* in different treatments were analyzed by RT‐qPCR. The results showed that the expression of *SikRbcS2* in *S. involucrata* seedlings growing for 3d under continuous white light was significantly higher than that growing for 3d under continuous dark by 6.28 times (Figure 2a), which suggested that light may regulate *SikRbcS2* expression. *SikRbcS2* expression increased rapidly and continuously after seedlings which grow in darkness transferring to light. After 3 hr of illumination, the expression of *SikRbcS2* was increased approximately 1.79 times as much as that in darkness. After 24 hr of illumination, the expression of *SikRbcS2* was increased approximately 4 times as much as that in darkness (Figure 2b). However, the expression of *SikRbcS2* was decreased rapidly after seedlings transferred to dark condition. After 3 hr of dark treatment, the expression of *SikRbcS2* was reduced to about 0.4 under light condition. During the whole process of white light turning into darkness, the expression of *SikRbcS2* showed an overall downward trend. After 24 hr of dark treatment, its expression was approximately reduced to 0.13 under light conditions (Figure 2c). These results suggested that light promoted the transcription of *SikRbcS2*, while darkness inhibited its transcription and was sensitive to light condition transformation.

Under long sunshine, the expression of *SikRbcS2* was increased rapidly after illumination, while that was decreased rapidly after darkness. In 24 hr, the expression of *SikRbcS2* in daytime was generally higher than that in night (Figure 2d). Light intensity often changes under natural light conditions, so changes in *SikRbcS2* expression levels under different light intensity were further detected. The results showed that in the selected light intensity ranges, the expression levels of *SikRbcS2* were increased significantly with the increase of light intensity (Figure 2e). In addition, *SikRbcS2* expression had tissue expression specificity. The expression of *SikRbcS2* in the over ground part of *S. involucrata* was significantly higher than that in the underground part. In addition, the expression of *SikRbcS2* in above ground organs under long sunlight was also significantly higher than that in darkness (Figure 2f). The above results showed that *SikRbcS2* was highly expressed by light and could change with the physiological rhythm of plants, the tissue expression was specific.

The *S. involucrata* was treated at different temperatures. The results showed that, with the decrease of stress temperature, the expression of *SikrbcS2* in seedling leaves of *S. involucrata* was increased gradually, which was 3.5 times higher than the control level at 8°C, and reached the highest level at 4°C with 5.2 times higher than the control level. Subsequently, the expression of *SikrbcS2* gene was decreased at 0°C, but it was still higher than the control level (Figure 2g). Under the cold treatment condition of 4°C, the expression of *SikrbcS2* gene was increased rapidly, reaching the highest level at 12 hr with 6.4 times higher than the control group (Figure 2h). This indicated that the expression of *SikrbcS2* gene was induced by low temperature. After soaking *S. involucrata* in 20% PEG6000 and 150 mmol NaCl for 24 hr, it was found that the expression of *SikrbcS2* gene was increased rapidly under simulated drought conditions. However, it was decreased after 3 hr and increased slowly after 12 hr (Figure 2i). Furthermore, the response to salt stress was different. Its expression reached the highest level at 3 hr and then was decreased rapidly (Figure 2j). These results indicated that the expression of *SikrbcS2* gene responded to low temperature, drought, and salt stress. This was consistent with the relevant functional elements of the promoter sequence.

### 3.3 Generation of transgenic tomato plants expressing *SikRbcS2*

**FIGURE 3:**
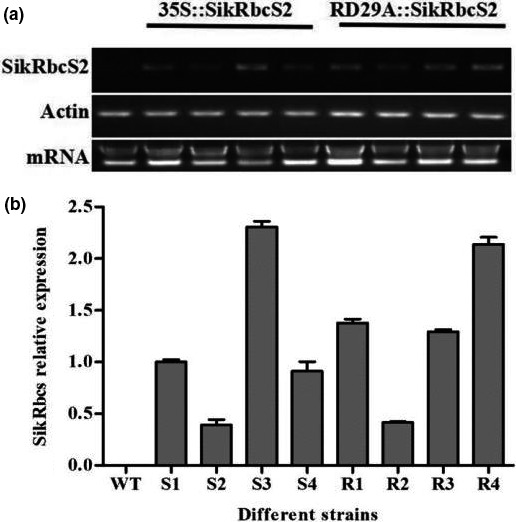
Expression levels of *SikRbcS2* in wild‐type and *SikRbcS2* overexpressing transgenic tomato plant lines. (a) Semi quantitative PCR of *SikRbcS2*‐overexpressing tomato plants. *SikRbcS2* mRNA levels of WT and *SikRbcS2*‐overexpressing plants. (b) Identification of *SikRbcS2*‐overexpressing tomato plants by qRT‐PCR. Transcript levels of *SikRbcS2* in WT and *SikRbcS2*‐overexpressing plants

15 strains of 35S::*SikRbcS2* and 12 strains of RD29A::*SikRbcS2* independent kanamycin‐resistant transgenic plants (T0 generation) were established, and PCR analysis confirmed that SikRbcs2 gene was successfully introduced into tomato plants. 8 transgenic plants transfected with 35S::SikRbcs2 and 7 RD29A::SikRbcs2 were obtained by PCR amplification using *SikRbcS2* primers. Among these transgenic plants, 4 strains had kanamycin resistance, respectively, with 3:1 of separation ratio in the T1 generation (Figure 3a). These 8 strains were selected for RT‐PCR and qRT‐PCR analysis. Compared with WT tomato, the relative mRNA levels of SikRbcs2 of transgenic plants S3 and R4 were 2.3 and 2.1 times than selected these two functional analyses, respectively (Figure 3b).

### 3.4 Effect of Rubisco and Rubisco activate on *SikRbcS2*‐overexpression tomato and WT tomato at low temperature

**FIGURE 4:**
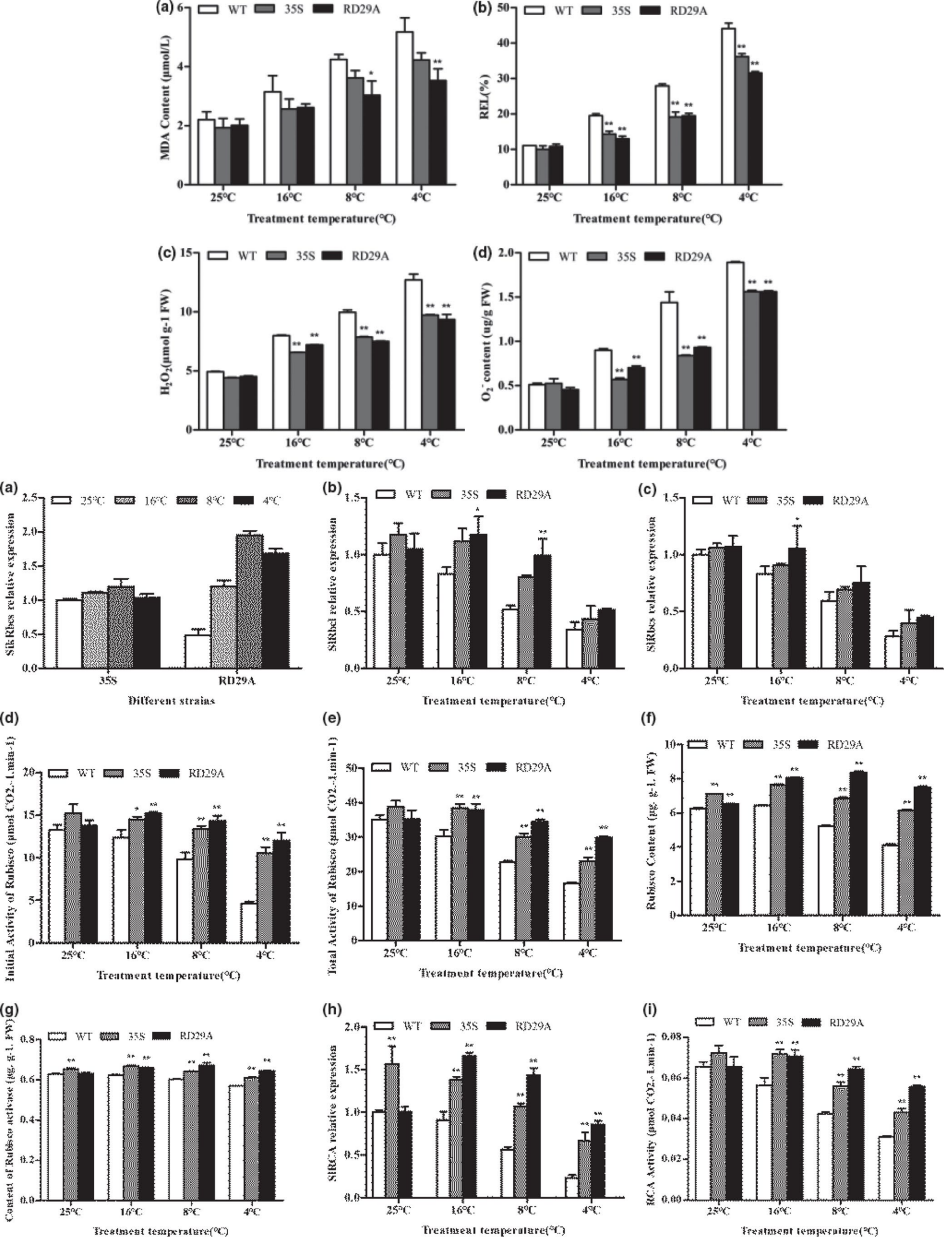
Effect of Rubisco and Rubisco activase on *SikRbcs2*‐overexpression tomato and wild‐type tomato at low temperature (a) The expression of *SikRbcs* in transgenic tomatoes was under low‐temperature stress. (b) Effects of low‐temperature stress on RbcL mRNA abundance in overexpression transgenic and wild‐type tomato seedlings．(c) Effects of low‐temperature stress on RbcS mRNA abundance in overexpression transgenic and wild‐type tomato seedlings．(d) Effects of low‐temperature stress on initial Rubisco activity in overexpression transgenic and wild‐type tomato seedlings．(e) Effects of low‐temperature stress on total Rubisco activity in overexpression transgenic and wild‐type tomato seedlings．(f) Effects of low‐temperature stress on Rubisco content in overexpression transgenic and wild‐type tomato seedlings．(g) Effects of low‐temperature stress on content of Rubisco activase in overexpression transgenic and wild‐type tomato seedlings．(h) Effects of low‐temperature stress on SlRCA mRNA abundance in overexpression transgenic and WT tomato seedlings．(i) Effects of low‐temperature stress on RCA activity in overexpression transgenic and WT tomato seedlings. Data are expressed as means ± *SD* (*n* = 3; *p < .05; **p < .01)

Before low‐temperature stress, the relative mRNA expression level of *SikRbcs* in 35s transgenic tomato was 52% higher than that in rd29a transgenic tomato (Figure 4a). With the decrease of temperature, the mRNA expression level of 35s transgenic changed little, but the expression level of rd29a transgenic gene was increased significantly, this indicated that the obtained transgenic lines were stable and reliable. Rubisco, a key enzyme in the Calvin cycle, consists of two subunits, RbcL and RbcS. It can be seen from Figure 4b and c that the overexpression of *SikRbcs* can significantly increase the mRNA expressions of RbcL in transgenic plants of 35s and rd29a. The mRNA expression levels of RbcL in 35s and rd29a were increased by 17% and 5%, respectively, in comparison with WT. Before low‐temperature stress and after low‐temperature stress, the mRNA expression levels of RbcL in each treatment were significantly decreased, but the mRNA expression levels of RbcL in 35s and rd29a were still significantly higher than WT with a low degree of decline. *SikRbcs2* overexpression also increased the mRNA expression of RbcS, and the variation trend was similar to that of the RbcL before and after high‐temperature stress. RbcL is encoded by chloroplast gene and plays a catalytic role. RbcS is encoded by nuclear gene and has the function of regulating Rubisco activity. According to Figure 4d and e, the introduction of exogenous *SikRbcs2* gene increased the relative expression level of Rubisco RbcL, which directly led to the increase of Rubisco activity in the case of little change of tomato's own RbcS gene in transgenic plants. The initial and total Rubisco activities of 35s and rd29a were increased by 14.9%, 4% and 10%, 0.3%, respectively, in comparison with WT. The activity of Rubisco was decreased significantly with the increase of low‐temperature stress, but the degree of reduction of Rubisco was significantly lower than that of WT plants. It suggested that the expression of *SikRbcs* at low temperature can maintain high photozyme activity in tomatoes, which may be an important mechanism to increase cold resistance. We measured Rubisco content and content of Rubisco activase in transgenic and WT plants. It was found that the contents of Rubisco and Rubisco activase in 35s were significantly higher than those in WT, and Rd29a was slightly higher than WT. Rubisco content and Rubisco activase of 35s and rd29a were significantly increased after low‐temperature treatment. Meanwhile, RCA expression was analyzed and it was found that compared with WT, the mRNA expression of 35s and rd29a was increased by 57% and 0.7%, respectively, before low‐temperature stress. After low‐temperature stress, mRNA expression levels of RCA in all treatments were significantly decreased. However, the mRNA expression levels of 35s and rd29a were still significantly higher than WT. As shown in Figure 4h and i, the changes in the activity of transgenic RCA were consistent with the changes in the relative mRNA expression levels of the corresponding RCA.

### 3.5 Effect of low temperature on chlorophyll fluorescence and photosynthetic parameters in WT and transgenic seedlings

**FIGURE 5:**
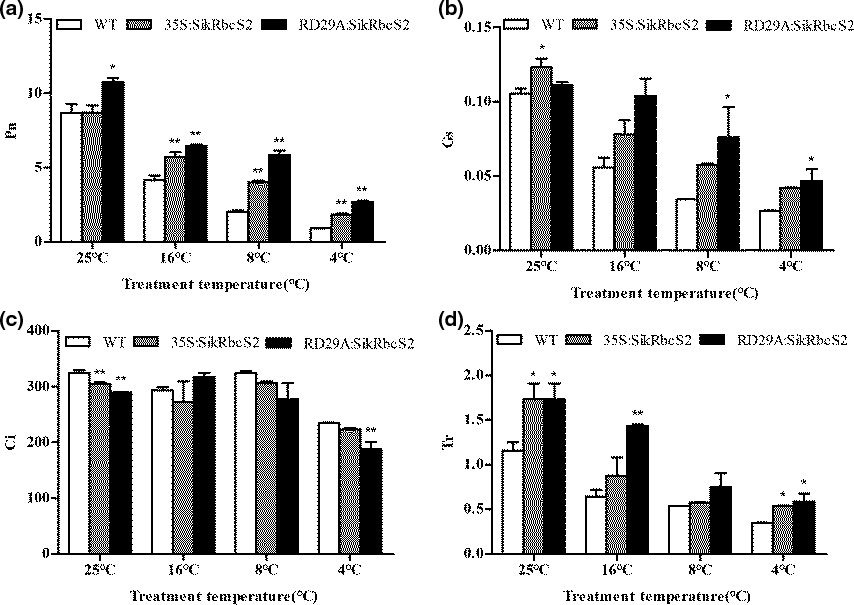
The effect of low‐temperature stress on Pn, Gs, Ci, and Tr values of WT and transgenic *SikRbcS2* tomatoes. (a) Pn values. (b) Gs values. (c) Ci values. (d) Tr values. Data are expressed as means ± *SD* (*n* = 3; *p < .05; **p < .01)

Low‐temperature stress was found to lead to significant decreases in Pn, but the extent of decrease varied among treatments. After treatment at 4°C for 5d, Pn was decreased by 79% and 75% in *35S*:*SikRbcS2* and *RD29A*:*SikRbcS2*, respectively, and decreased by 89% in WT plants. Afterward, Pn was continually decreased in both transgenic and WT lines when the duration of the exposure to low‐temperature stress was increased, but the decrease in Pn was more obvious in WT than in transgenic plants (Figure 5a). The Gs and Tr values of transgenic and WT leaves were found to both decrease in response to longer and more severe low‐temperature stress, whereas Ci values were increased as the time under stress increased. This suggested that the decrease in Pn under low‐temperature stress may be associated with nonstomatal limitation. Compared to WT plants, the transgenic plants showed greater Gs and Tr values, but lower Ci values during low‐temperature stress. These results suggested that the overexpression of *SikRbcS2* played a significant role in alleviating injuries to the photosynthetic apparatus caused by low temperatures, ostensibly by maintaining higher photosynthetic activity in tomato seedling mesophyll cells (Figure 5b–d).

### 3.6 The effect of low temperature on chlorophyll pigment content in WT and transgenic seedlings

**FIGURE 6:**
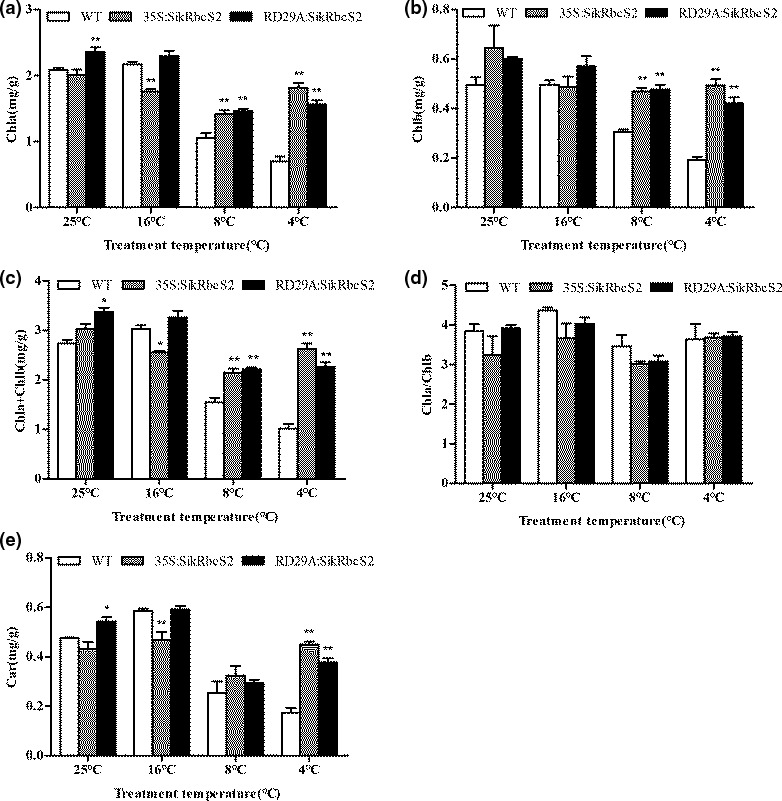
The effect of low‐temperature stress on the chlorophyll contents of WT and transgenic seedlings. (a) Chla content of plants. (b) Chlb content of plants. (c) (Chla + Chlb) content of plants. (d) Chla/Chlb content of plants. (e) Car content of plants. Data are expressed as means ± *SD* (*n* = 3; *p < .05; **p < .01). (a–d) Car content of plants

Next, we measured the content of chlorophyll a, chlorophyll b, and carotenoids, three major pigments involved in plant photosynthesis. We found that relative to WT seedlings, seedlings from the two transgenic lines of tomato showed lower reductions in chlorophyll content in response to low‐temperature stress treatments (Figure 6a–c). This finding was consistent with the morphology of these plants. Taken together, this finding suggested that transgenic tomato plants expressing *SikRbcS2* had better low‐temperature stress resistance than WT tomato plants. We found no significant changes in the chlorophyll a/b (Chla/Chlb) ratio among the WT and transgenic seedlings (Figure 6d). Moreover, the carotenoid (Car) content of WT tomato seedlings also fell sharply (i.e., at 4°C it had fallen by 64% compared to pretreatment levels). In contrast, the carotenoid levels of 35S:*SikRbcS2* tomatoes fell slowly from 25°C to 8°C and then rose again at 4°C. A similar pattern was found with RD29A:*SikRbcS2* seedlings. The decline in photosynthetic pigments contents meant that at low temperatures, the photosynthetic capacity of the transgenic tomato lines was higher than that of WT tomato seedlings (Figure 6e).

### 3.7 Physiological analysis and accumulation of ROS in wild‐type and *SikRbcS2*‐overexpressing transgenic tomato plant lines under cold stress

**FIGURE 7:**
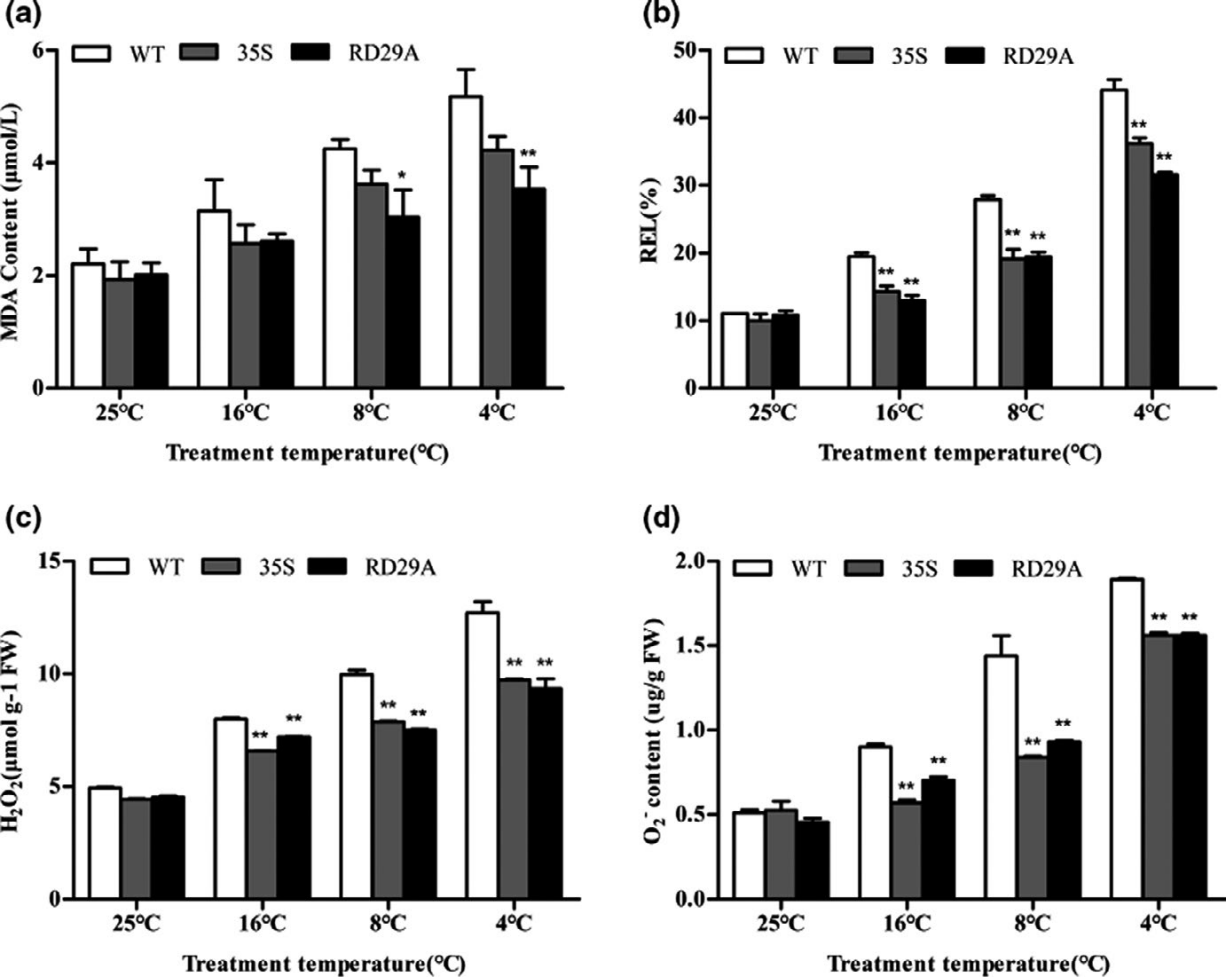
Physiological analysis and accumulation of ROS in wild‐type and *SikRbcS2*‐overexpressing transgenic tomato plant lines under cold stress. (a) MDA content. (b) Relative electrolyte leakage. (c) H_2_O_2._ (d) O_2_^−^. Data for the WT and transgenic plants are expressed as means ± *SD* of three replicates. Asterisk (s) indicate significant difference between WT and transgenic plants; *p < .05 and **p < .01

Under normal conditions, there was no significant difference in REL and MDA contents between transgenic and WT plants. With the decrease of temperature, the REL and MDA contents of leaves of both WT and transgenic plants were gradually increased, but the content of transgenic plants was smaller than that of WT plants (Figure 7a and b). These results showed that the overexpression of *SikRbcS2* reduced the damage of low‐temperature stress to plant cell membrane. We examined the levels of H_2_O_2_ and O_2_− in the leaves of plants. Under normal growth conditions, transgenic plants were the same as WT plants. There was very little accumulation of H_2_O_2_ and O_2_− in the leaves. As the treatment temperature decreased, the accumulation of H_2_O_2_ and O_2_− in the leaves of overexpressing transgenic plants and WT plants were increased. Compared with WT, the accumulation of H_2_O_2_ and O_2_− in the leaves of transgenic lines was significantly less (Figure 7c and d). Excessive accumulation of ROS in the leaves of WT plants will cause oxidative stress, cell damage, and even death.

## 4 DISCUSSION

Our analysis of chloroplast pigment content found that the contents of Chla, Chlb, and Chla + Chlb of the transgenic *SikRbcS2*‐carrying tomato lines were significantly higher than those of WT tomato, while the Chla/Chlb values of all lines did not significantly differ. These results showed that transgenic tomato was more tolerant to low‐temperature stress. At the same time, the carotene content of the transgenic tomato lines was significantly higher than that of WT tomato when all plants were subjected to low‐temperature stress treatments. This pigment is related to heat dissipation and can help plants to dissipate extra light energy to resist damage caused by light. The presence of higher carotene content in the transgenic tomato lines indicated that these lines had better photosynthetic protection and maintained higher linear electron transport capacity at low temperatures.

Chlorophyll fluorescence parameters reflect light energy absorption and the light utilization of plant leaves (Maxwell & Johnson, 2000). The photosynthetic pigments in the optical system can be destroyed during exposure to low temperatures, resulting in decreased photo‐absorption capacity of the optical system (PSI and PSII) and eventually to decreased photosynthetic capacity (Kooten & Snel, 1990). The light energy absorbed by chlorophyll is converted into chlorophyll fluorescence. The measurement of chlorophyll fluorescence is an effective alternative to the measurement of light absorption capacity of plant leaves under adversity (Krause & Weis, 1984). The effects of low‐temperature stress on photosynthesis in plants are multifaceted and involve not only directly causing damage to the photosynthetic apparatus, but also affecting the enzymes involved in photosynthetic electron transport, photosynthetic phosphorylation, and dark reactions (Schreiber, Bilger, & Neubauer, 1995). In this study, we found that under low‐temperature stress, the fixed fluorescence F0 of chlorophyll was increased. We also found that ERF and the ratios of Fv/Fo and Fv/Fm were significantly reduced. Taken together, these data suggested that the PSII reaction center was inactivated. Low‐temperature stress reduced the ability of plants to utilize light energy. The analysis of chlorophyll fluorescence parameters showed that, in response to low‐temperature stress conditions, *SikRbcS2*‐carrying transgenic lines of tomato showed greater leaf PSII photochemical efficiency (Fv/Fm) and PSII activity (Fv/Fo) than WT tomato plants. We also found that transgenic tomato plants showed gradual decreases in qP and ETR values, and at each temperature point, the transgenic lines’ values were significantly higher than those of WT tomatoes. Taken together, these results indicated that transgenic tomatoes can maintain higher photosynthetic electron transfer efficiency and generate a greater isomerization force to meet the demand of dark reaction processes. The reduction in Pn caused by abiotic stress is attributed to an inhibition of Rubisco activity. All tomato lines showed that reduced Pn as the low‐temperature stress treatments became more extreme. At 16°C and 4°C, we found that the Pn values of the transgenic tomatoes were significantly higher than those of the WT tomatoes. These results suggested that the photosynthetic mechanisms of the transgenic tomatoes were less damaged by exposure to low‐temperature treatments than those of the WT tomatoes.

During photosynthesis, stomata are windows for the exchange of gases, including H_2_O and CO_2_ (Farquhar & Sharkey, 1982; Wong, Cowan, & Farquhar, 1979). The change of stomatal conductance to CO_2_ will cause changes in intercellular CO_2_ concentration (Ci), which can thereby affect the photosynthetic rate (Ainsworth & Rogers, 2007). Low temperatures may decrease the photosynthetic rate by reducing stomatal conductance (Gs) and restricting the entry of external CO_2_ into cellular spaces via stomata (Delucia, 1986). We found that all tomato lines showed reduced GS as the low‐temperature stress treatments became more extreme. Between 25°C and 8°C, we found no significant differences between WT and transgenic tomato lines with respecting to Ci. However, at 4°C, the intercellular CO_2_ concentration fell sharply. This indicated that under low‐temperature stress, inhibition of stomatal system affected the plant absorption of CO_2_ and thus inhibited photosynthesis. We showed that the transpiration rate of transgenic tomatoes decreased as the stress treatments reached lower temperatures.

When plants are under stress, reductions in the net photosynthetic rate are often caused by two main factors, stomatal and nonstomatal factors (Augé, Toler, & Saxton, 2015; Nilsen & Orcutt, 1996). When the closure of stoma causes reduction of intercellular CO_2_ concentration, the result is a reduced net photosynthetic rate (Radin, Parker, & Guinn, 1982). However, when the net photosynthetic rate and stomatal conductance are decreased together, the intercellular CO_2_ concentration is increased, that is, the reduction of net photosynthetic rate was likely caused by nonstomatal factors. In this experiment, the net photosynthetic rate was decreased due to stomatal closure, which led to reductions in intercellular CO_2_ concentration, thereby reducing the photosynthetic capacity. Thus, we concluded that the reduced photosynthetic capacity was caused by stomatal factors.

The RD29A promoter is cold‐induced. In this experiment, cold‐induced expression of *SikRbcS2* genes in tomato plants subjected to low‐temperature stress treatments (Kasuga, Miura, Shinozaki, & Yamaguchi‐Shinozaki, 2004). It resulted in the values of Fv/Fm, ETR, qP, and qN being similar to those of the transgenic lines which constitutively expressed *SikRbcS2*. Here, we observed that the *SikRbcS2* gene notably improved the low‐temperature stress response of tomato plants. However, whether this effect is due to changes in the structure or function of Rubisco is not clear, and it should be a focal point of future studies of *SikRbcS2*.

5

**TABLE 2:** Functional analysis of cis‐elements of *SikrbcS2* promoter

Regulatory sequence	Sequence	Biological function
A‐box	CCGTCC	cis‐acting regulatory element
AE‐box	AGAAACAA	Part of a module for light response
ATCT‐motif	AATCTAATCT	Part of a conserved DNA module involved in light responsiveness
AuxRE	TGTCTCAATAAG	Part of an auxin‐responsive element
CAT‐box	GCCACT	cis‐acting regulatory element related to meristem expression
CCGTCC‐box	CCGTCC	cis‐acting regulatory element related to meristem specific activation
GA‐motif	AAAGATGA	Part of a light responsive element
GAG‐motif	AGAGAG/T	Part of a light responsive element
HSE	AGAAAATTCG	cis‐acting element involved in heat stress responsiveness
I‐box	GATATGG	Part of a light responsive element
MBS	T/CAACTG	MYB binding site involved in drought‐inducibility
O2‐site	GATGATGTGG	cis‐acting regulatory element involved in zein metabolism regulation
P‐box	GCATTTTGAGT	Gibberellin‐responsive element
Skn‐1_motif	GTCAT	cis‐acting regulatory element required for endosperm expression
TATA‐box	TATA	Core promoter element around ‐30 of transcription start
TC‐rich repeats	ATTCTCTAAC	cis‐acting element involved in defense and stress responsiveness
TGACG‐motif	TGACG	cis‐acting regulatory element involved in the MeJA‐responsiveness
ABRE	CCTACGTGGC	cis‐acting element involved in the abscisic acid responsiveness
ARE	TGGTTT	cis‐acting regulatory element essential for the anaerobic induction
LER	CCGAAA	cis‐acting element involved in low‐temperature responsiveness
ELI‐box3	AAACCAATT	Elicitor‐responsive element
G‐Box	CACGTT	cis‐acting regulatory element involved in light responsiveness
GARE‐motif	AAACAGA	Gibberellin‐responsive element
GC‐motif	CCCCCG	Enhancer‐like element involved in anoxic specific inducibility
GCN4_motif	TGTGTCA	cis‐regulatory element involved in endosperm expression
Sp1	GGGCGG	Light responsive element
TCT‐motif	TCTTAC	Part of a light responsive element
TGA‐element	AACGAC	Auxin‐responsive element
Circadian	CAANNNNATC	cis‐acting regulatory element involved in circadian control

**TABLE 3:** The effect of low‐temperature stress on the fresh (FW) and dry weights (DW) of shoots and roots of WT and *SikRbcS2* tomato plants

Treatment			FW (g)	DW (g)
Shoot	25°C	WT	28.96 ± 2.78	3.35 ± 0.37
		*35S:SikRbcS2*	34.42 ± 1.79	3.53 ± 0.19
		*RD29A:SikRbcS2*	31.89 ± 3.0	3.5 ± 0.45
	16°C	WT	29.76 ± 1.32	3.47 ± 0.12
		*35S:SikRbcS2*	35.82 ± 2.39	3.73 ± 0.28
		*RD29A:SikRbcS2*	33.69 ± 2.95	3.75 ± 0.27
	8°C	WT	28.06 ± 3.12	3.41 ± 0.31
		*35S:SikRbcS2*	35.87 ± 1.81	3.75 ± 0.29
		*RD29A:SikRbcS2*	33.71 ± 2.92	3.78 ± 0.22
	4°C	WT	23.26 ± 2.83	2.91 ± 0.26
		*35S:SikRbcS2*	34.57 ± 3.75	3.45 ± 0.29
		*RD29A:SikRbcS2*	31.51 ± 2.09	3.58 ± 0.22
Root	25°C	WT	6.28 ± 1.33	0.89 ± 0.18
		*35S:SikRbcS2*	6.7 ± 0.37	1.09 ± 0.19
		*RD29A:SikRbcS2*	6.6 ± 0.16	1.05 ± 0.30
	16°C	WT	7.18 ± 0.65	0.97 ± 0.05
		*35S:SikRbcS2*	7.9 ± 0.73	1.19 ± 0.19
		*RD29A:SikRbcS2*	8.11 ± 0.30	1.21 ± 0.26
	8°C	WT	7.14 ± 0.24	0.95 ± 0.27
		*35S:SikRbcS2*	7.92 ± 0.34	1.18 ± 0.23
		*RD29A:SikRbcS2*	8.1 ± 0.35	1.23 ± 0.21
	4°C	WT	4.84 ± 0.29	0.92 ± 0.34
		*35S:SikRbcS2*	6.02 ± 0.24	1.16 ± 0.19
		*RD29A:SikRbcS2*	6.1 ± 0.23	1.22 ± 0.21
